# Stability, Speed, and Constraints for Structural Coarse-Graining in VOTCA

**DOI:** 10.1021/acs.jctc.2c00665

**Published:** 2023-01-11

**Authors:** Marvin P. Bernhardt, Martin Hanke, Nico F.A. van der Vegt

**Affiliations:** † Eduard-Zintl-Institut für Anorganische und Physikalische Chemie, Technische Universität Darmstadt, Alarich-Weiss-Straße 10, 64287Darmstadt, Germany; ‡ Institut für Mathematik, Johannes Gutenberg-Universität Mainz, Staudingerweg 9, 55128Mainz, Germany

## Abstract

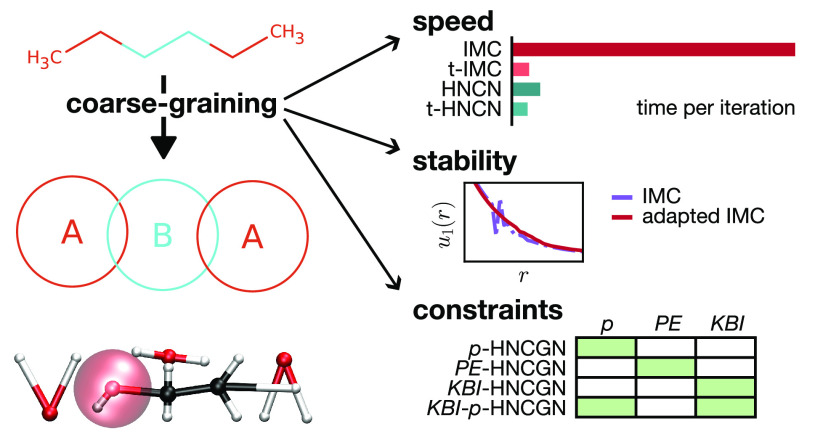

Structural coarse-graining involves the inverse problem of deriving pair potentials that reproduce target radial distribution functions. Despite its clear mathematical formulation, there are open questions about the existing methods concerning speed, stability, and physical representability of the resulting potentials. In this work, we make progress on several aspects of iterative methods used to solve the inverse problem. Based on integral equation theory, we derive fast Gauss–Newton schemes applicable to very general systems, including molecules with bonds and mixtures. Our methods are similar to inverse Monte Carlo in terms of convergence speed and have a similar cost per iteration as iterative Boltzmann inversion. We investigate stability problems in our schemes and in the inverse Monte Carlo method and propose modifications to fix them. Furthermore, we establish how the pair potential can be constrained at each iteration to reproduce the pressure, Kirkwood–Buff integral, or the enthalpy of vaporization. We demonstrate the potential of our approach in deriving coarse-grained force fields for nine different solvents and their mixtures. All methods described are implemented in the free and open VOTCA software framework for systematic coarse-graining.

## 1 Introduction

Coarse-grained molecular simulations present the challenge of representing a molecular system with a large number of atomistic degrees of freedom by a simple model with a significantly reduced number of degrees of freedom. Its main applications lie in polymer and biomolecular sciences where the time and length scales are out of reach for atomistic molecular dynamics (MD) simulations.^1−4^ An important class of coarse-grained (CG) models is particle-based, with pair potentials realizing repulsion and attraction between groups of atoms combined into so-called beads. Several methods exist to obtain CG pair potentials: Top-down methods parametrize the CG potentials based on experimental data.^5^ Bottom-up methods derive potentials from some reference atomistic model with a preexisting force field or ab initio MD. Examples for the latter are relative entropy minimization (REM) and force-matching.^6,7^ Machine learning algorithms can be trained to yield potentials that reproduce, e.g., force-matching results.^8^ Two methods, inverse Monte Carlo (IMC) and iterative Boltzmann inversion (IBI), explicitly optimize pair potentials to match the radial distribution function (RDF) of the mapped atomic reference.^9,10^ The RE method has been proven to be equivalent to IMC if pair potentials are optimized by a Newton scheme.^11,12^

The mathematical classification of the structural coarse-graining problem is that of an inverse problem. Only a complicated forward function from the potential to the RDF exists (the MD simulation), but the opposite direction is of interest.^13^ The Henderson theorem states that the potential for a given RDF is unique up to a constant.^14,15^ IBI seems to converge to the RDF, but the potential is still changing even after thousands of iterations.^16^ Its use is nevertheless prevalent due to its very simple “one-line” implementation. IMC on the other hand, i.e., the Newton iteration, while in theory the better choice, is not without its problems. Sampling the cross-correlations of particle counts for the IMC update is computationally expensive. Even though it is more rigorous than IBI, it has stability issues as it sometimes produces noisy potentials. Regularization, scaling of the potential update, or preiterations of IBI are therefore often employed.^17,18^ Despite these fixes, it seems that it is not understood why IMC has instabilities that IBI does not have.

In two previous papers, we have introduced a new type of structural coarse-graining method for solving the inverse problem which is based on integral equation theory.^19,20^ It uses the hypernetted-chain (HNC) equation for an approximate relation between RDF and the potential. The new iteration methods lead to convergence in a similar number of iterations as IMC while requiring no sampling of cross-correlation matrices. So far, our method was only available for systems of single beads. General systems with multiple beads and bonds can be treated with the Reference Interaction Site Model (RISM) equation,^21^ based on which we generalize our method. We also make use of symmetry reduction of the RISM equations that arise when multiple beads in a molecule are equivalent.^22^

Two of the significant challenges in coarse-graining are transferability across different state points and composition as well as representability in terms of thermodynamic properties such as pressure or compressibility. It has been argued that different additional terms are needed to achieve those.^23,24^ Examples are temperature-dependent potentials,^25^ local density-dependent potentials,^26^ and volume-dependent potentials.^24^ At the same time, it is unclear which properties can be represented well with pair potentials alone. Several thermodynamic properties can be expressed as a function of the RDF and the pair potential and therefore be constrained using a Gauss–Newton scheme.^20,27^ In this paper, we introduce three different thermodynamic constraints and test whether they can be combined.

The paper has the following structure: In Section 2.1, we first introduce the general scheme of the iterative methods used. In Sections 2.2–2.5, the new general integral equation ansatz to connect structure and potential is derived. In Sections 2.6–2.10, we introduce several modifications of the algorithm that address speed, thermodynamic consistency, and stability. In the remaining sections, we apply the methods to a neon–argon mixture, hexane, and various organic solvents. All methods and algorithms used in this paper have been implemented in Python and Numpy and integrated into the VOTCA software package.^28,29^ The code resides currently in the csg/multi-iie2 branch on https://github.com/votca/votca, but will be merged into the main branch soon. We also make all simulation files, RDFs, and potentials available at https://tudatalib.ulb.tu-darmstadt.de/handle/tudatalib/3492.

## 2 Theory

The problem we are solving is finding a set of potentials that reproduces a given set of RDFs. The target RDFs are typically obtained from a mapped atomistic trajectory, i.e., an atomistic trajectory analyzed in terms of the CG degrees of freedom. We assume the mapping scheme that relates the position of the CG beads to the atoms (usually the center of mass) is fixed. The iterative approach is to use an initial potential guess and then make repeated updates based on the remaining difference between the current and target RDFs.

### 2.1 Newton and Gauss–Newton Methods

For a system with different beads, we are interested in all potentials *u*_*αβ*_ between them, where α, β are indices for bead types. We stack all potentials to a single vector *u* = (*u*_*αα*_, *u*_*αβ*_, ...). For a system with *n*_t_ bead types, there are  interactions. Note that each potential is itself a tabulated potential *u*_*αβ*_ = (*u*_*αβ*1_, *u*_*αβ*2_, ..., *u*_*αβn*_cut__), and *u* can be seen as a single one-dimensional vector. The index *n*_cut_ equals the number of grid points up to the cutoff *r*_cut_. Similarly, we define the vector *g* from all *n*_i_ RDFs. In Newton’s method, the potentials are iteratively updated as
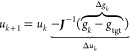
1The subscript *k* denotes that iteration *k* and *g*_*k*_ are the RDFs obtained from MD with potentials *u*_*k*_. Matrix ***J*** is the Jacobian with the elements
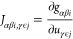
2Here, the indices *i* and *j* represent the distance between the interacting beads α, β and γ, ϵ, respectively. We note that eq 1 provides the explicit formulation of the potential update, but in practice, it is advantageous to solve the system of linear equations without inverting the matrix.

The Jacobian ***J*** is assumed to be square; i.e., the potential tables have as many data points as the RDFs used for the update. We relax this condition and optionally introduce a diagonal weighting matrix ***W*** and use a Gauss–Newton algorithm, where the update is computed as
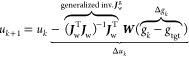
3with the weighted Jacobian ***J***_w_ = ***WJ***. Each RDF can now have more grid points than the potentials: *g*_*αβ*_ = (*g*_*αβ*1_, *g*_*αβ*2_, ... *u*_*αβn*_res__) with *n*_res_ ≥ *n*_cut_. This type of update minimizes the norm
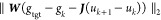
4In other words, the potentials are updated such that the weighted difference between the target RDFs *g*_tgt_ and the new expected RDFs *g*_*k*+1_ ≈ *g*_*k*_ + ***J***(*u*_*k*+1_ – *u*_*k*_) is minimized. This type of formulation has several advantages. First, it allows one to restrict the potential (update) to a cutoff *r*_cut_ while optimizing the RDF up to a longer (residuum-)cut *r*_res_. Optionally, the tail can be given a larger weight in the optimization by the weighting matrix. Second, the scheme allows for the optimization of a selection of RDFs by changing a similar or smaller subset of potentials. This selective scheme is introduced in Section 2.7. Third, constraints can be introduced, see Section 2.8. Before we explore those topics, we explain how the Jacobian can be obtained.

### 2.2 IBI and IMC

When the derivative in eq 2 is approximated by the corresponding derivative for the low-density approximation
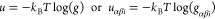
5the Jacobian becomes a diagonal matrix because the value of the pair potential *u*_*αβi*_ only depends on *g*_*k*,*αβi*_ at the same α, β, and *i*. One can show that^19^ the resulting low-density Newton scheme is numerically close to the IBI scheme^10^
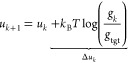
6which itself is not a Newton scheme. The low-density Newton scheme and the IBI method are local in two senses: (i) the interaction of two bead types is not influenced by the structure of a different pair of bead types (e.g., *Δu*_*k*,AA_ does not depend on *g*_*k*,AB_ or *g*_tgt,AB_), and (ii) the potential update at each distance only depends on the RDF mismatch at that distance (e.g., *Δu*_*k*,AA1_ does not depend on *g*_*k*,AA2_ or *g*_tgt,AA2_).

The inverse Monte Carlo method, on the other hand, is the full Newton scheme.^9^ IMC is expressed using the quantity *S*_*αβ*_, which describes the total number of bead pairs between bead types α and β. It is related to the RDF via , where *r*_*i*_ is the distance, *N*_α_ is the number of beads of type α, and *V* is the system volume. The factor (1 + δ_*αβ*_) arises because *S*_*αβ*_ is a measure for the number of pair interactions, and double-counting has to be avoided when α = β. For a system with pair potentials, the elements of the IMC Jacobian can be computed from the cross-correlations between the particle numbers *S*_*αβi*_ at different distances
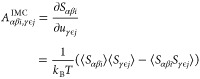
7Equation 7 contains no approximations and is only limited by sampling. The matrix ***A*** is the Jacobian in terms of *S* and *u*. The IMC Jacobian ***J***^IMC^ in terms of *g* and *u* is expressed as
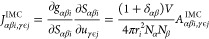
8

### 2.3 Integral Equation theory

Another way to obtain a connection between the potential and the RDF is integral equation theory. In particular, the Ornstein–Zernike equation defines the direct correlation function *c*_*αβ*_ between beads α and β, see Section 2.4. Together with a closure relation, one obtains a direct but approximate relation between the potential and the RDF. Note that we use translation-invariant integral equation theory and thereby assume a homogeneous system.

We use either the hypernetted-chain closure equation^30^
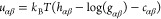
9or the Percus–Yevick closure equation
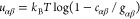
10Here, *h*_*αβ*_ = *g*_*αβ*_ – 1 is the total correlation function between bead types α and β. The direct correlation functions *c* can be obtained from the RDFs *g*, as explained in the next section. Equations 9 and 10 can be used as an initial guess for the potential, which offers a better starting point than the potential of mean force from eq 5 (often called Boltzmann inversion).^31^

For the use in Newton’s method or Gauss–Newton algorithm, the Jacobian is needed. From eqs 9 and 10 we can obtain an approximation to the inverse of the Jacobian by differentiation. The elements of the Jacobian inverse using the hypernetted-chain closure are
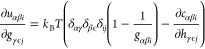
11We call the iterations based on this Jacobian HNCN or HNCGN, depending on whether Newton or Gauss–Newton is used. The derivative  has to be obtained independently, see Section 2.5. In principle, the Percus–Yevick closure can also be differentiated, but the implementation of this closure relation is less straightforward and, so far, untested. In this paper, we only consider the hypernetted-chain closure.

In eqs 9 and 10, the pair potential is assumed to be the total nonbonded pair potential between two beads; additional nonbonded potential terms, e.g., charges in the CG model, would prohibit the meaningful use of integral equation methods in the given form. On the other hand, bonded potential terms (bonds and angles) may be present in the system: They influence the given equations only indirectly via the intramolecular structure, as described in the next section.

In integral equation theory, the quantities *x*_*αβ*_ and *x*_*βα*_, where *x* can be *u*, *g*, *h*, ω, or *c*, are distinguished (even though they are usually equal). This is not the case outside of Sections 2.3–2.5, where only *x*_*αβ*_ is considered. This detail has subtle implications on how to use the results from eqs 9 to 11: To use the potential from eqs 9 and 10 in an MD simulation, *u*_*αβ*_ and *u*_*βα*_ are averaged. The Jacobian (inverse of eq 11) initially contains derivatives with respect to *u*_*ϵγ*_ and *u*_*γϵ*_. Those elements are summed up and reduced to one element. Elements that describe the derivatives of *g*_*αβ*_ and *g*_*βα*_ are averaged, and one of them is removed, such that the Jacobian is square again.

### 2.4 Direct Correlation Function

To evaluate the above equations, we need the direct correlation function and its derivative. The Ornstein–Zernike (OZ) equation defines the direct correlation function. If there are any bonds between the beads, the Reference Interaction Site Model (RISM) equation has to be used, which can be seen as a generalization of the OZ equation.^21,30^ The OZ equation and the RISM equation are matrix equations for systems with different bead types. Therefore, here we introduce a new arrangement of the RDF data: a matrix ***g*** with elements *g*_*αβ*_ in row α and column β. Each element can again be a function of the distance *r*, but in this section, there will be mostly Fourier transformed variables and then each element is a function of the wave vector.

Not considering any indistinguishability between beads in a molecule, the RISM theory is written as a matrix equation
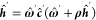
12Here, ***ĥ***′ and ***c*^**′ are matrices of the total and direct correlation function, respectively. The matrix **ω̂**′ contains the intramolecular density functions. The hats denote the three-dimensional Fourier transform of the radially symmetric functions.^19^ By using the Fourier transform, the convolution that takes place in real space becomes a multiplication, and the matrix equation can be solved for each wave vector independently. The matrices are square with dimension *n*_td_, the number of beads per molecule. For example, *n*_td_ is 12 for benzene in atomistic resolution. Inputs into the equation are the total correlation functions *h* and the intramolecular density functions ω. For an initial guess, both can be obtained from the reference system (*h*_tgt_, ω_tgt_).

Some beads might be equivalent, considering the bead type and symmetry of the molecule. This equivalence allows for a similarity transformation to a symmetry-reduced RISM equation as derived by Bertagnolli et al.^22^ The needed transformation matrix ***T*** is block-diagonal with *n*_t_ blocks where *n*_t_ is the number of different bead types. Each block ***T***^α^ is an *n*_α_ × *n*_α_ matrix where *n*_α_ is the number of indistinguishable beads of type α. For example, *n*_t_ = 2, *n*_C_ = 6, and *n*_H_ = 6, for benzene in atomistic resolution. The elements of the first column of ***T***^α^ all equal *n*_α_^–1/2^. The other columns of ***T***^α^ form an orthogonal basis such that each block ***T***^α^ is an orthonormal matrix. We obtain the transformed matrices
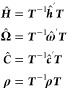
13The matrix **ρ** is a diagonal matrix with the densities of each bead unaffected by the similarity transformation. Inserting these definitions in eq 12 and using ***T***^–1^***T*** = ***TT***^–1^ = ***I***_*n*_td__, this transformation reduces the RISM-OZ equation to
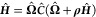
14In any block ***C*^**^*αβ*^ of ***C*^**, which corresponds to two bead types α and β, only one element is nonzero. The same is true for ***H*^** and **Ω̂**. Thereby the dimensionality is effectively reduced from *n*_td_ in eq 12 to *n*_t_ in eq 14, and the empty rows and columns can be omitted (we from now on treat those matrices as *n*_t_ × *n*_t_). For further details on symmetry reduction, we refer to the original paper.^22^

Note that commonly the analysis of the trajectory will average over indistinguishable beads when calculating distribution functions. Therefore, one does not obtain ***h***′ but ***h***. To obtain ***H***, the explicit transformation with ***T*** is therefore not needed. In the appendix, we give the factors needed to convert between ***h***, ***c*** and ***H***, ***C*** and clarify how **Ω** is calculated.

Inverting eq 14, we obtain the adapted direct correlation function from the total correlation and intramolecular density function.
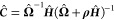
15Afterward, the direct correlation function matrix ***c*^** is obtained by the inverse of the prefactor from eq 45 in the Appendix.

### 2.5 Direct Correlation Function Derivative

For the derivative of the HNC eq 11, we need the derivative of the direct correlation function with respect to the total correlation function. When using the symmetry-adapted RISM we first have to relate the derivative to the adapted variables. Using the relation between the elements of ***C*** to ***c*** and ***H*** to ***h*** from eq 45 the following holds
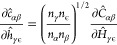
16To obtain the derivative on the right-hand side of eq 16, we start from eq 15 for ***C*^** = ***C*^**(***H*^**) and add a perturbation Δ***H*^** to ***H*^** to define
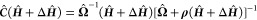
17This expression is transformed in several steps, writing **Ω̂** + ***ρH*^** as ***Z*** for brevity and ignoring higher order terms
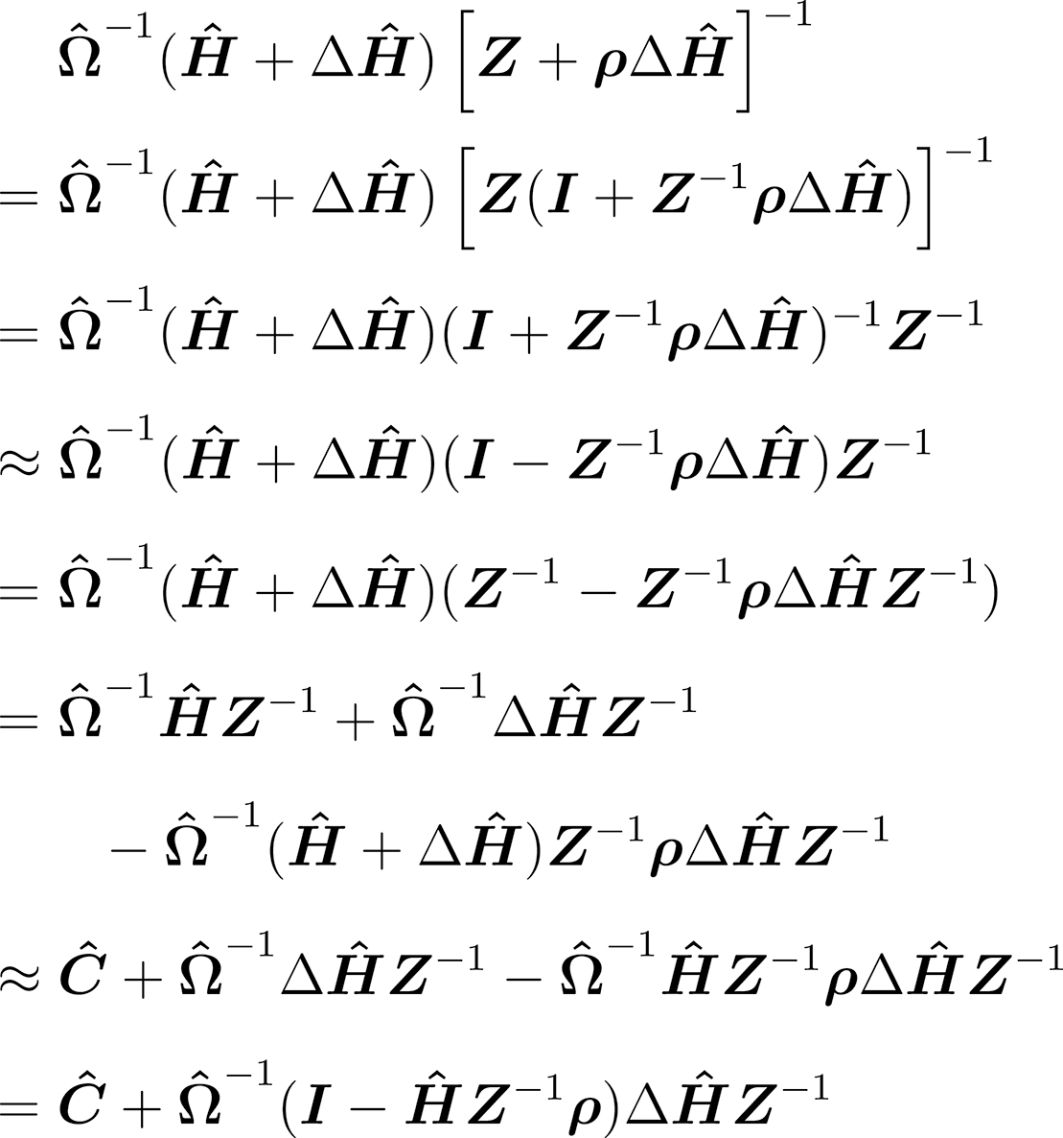
and finally
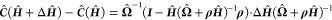
18We apply vectorization to eq 18, which transforms a matrix into a long vector going through the elements column-wise. Using the identity vec(***UVW***) = (***W***^T^ ⊗ ***U***) vec(***V***), where ⊗ is the Kronecker product, we eventually arrive at the derivative
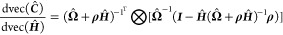
19This derivative reduces to eq 9 from our previous paper^20^ for the single bead case. The distributions ***H*** and **Ω** are obtained from the MD simulation with the current potential, except when the technique described in the next section is used.

Using eqs 16 and 19, we obtain the derivatives , which is a numeric value for each wave vector. To obtain the same derivative in real space, as needed for eq 11, we use
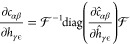
20where  is the Fourier matrix. The derivative  is now a matrix with elements .

### 2.6 One-Time Jacobian Calculation

The IMC and HNC methods are more expensive per iteration than IBI because the Jacobian matrix has to be calculated. To make those methods faster, one can attempt to calculate it only once.

The averages in eq 7 are evaluated in the system with the current potential guess. A computation-intensive sampling of long MD trajectories has to be carried out at every iteration. A simple shortcut is to only calculate it once at the target using the trajectory of the reference simulation, provided the latter is available
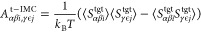
21where the brackets denote in this case an average over the fine-grained trajectory. Equation 21 contains an approximation to the Jacobian, because the IMC formalism assumes only pairwise interactions. The configurations in the mapped atomistic trajectory are, however, determined by the multibody potential of mean force (mPMF), which is only approximately decomposable in pair potentials. The approximation could lead to instabilities or slow convergence if the mPMF decomposes poorly into pair potentials, but this was not tested systematically. It is also important that the sampling of eq 21 is evaluated in the NVT ensemble, not in the NpT ensemble. The reason is that under NpT conditions, the barostat changes the probabilities for multiple interactions being at a certain distance by scaling the box volume. We prepend a “t” and write t-IMC to denote that the IMC matrix was calculated at the target configurations.

A similar shortcut can be taken for the HNC Jacobian as described in eq 11. In particular, we have implemented the option to calculate  only once from the target distributions. As described in our last paper, this derivative contains artifacts if not calculated from an RDF with double length.^20^ When  is calculated only once at the very beginning, then it is sufficient to calculate the RDF on the short distance range of the potential in all subsequent iterations. This leads to a considerable performance improvement since the computational cost of calculating the RDF grows rapidly with the maximum pair distance. We prepend a “t” and write t-HNCN or t-HNCGN if the derivative  is calculated only once from the target RDF.

### 2.7 Selective Optimization

In Section 2.1, we explained that the Gauss–Newton method, by default, minimizes the norm ∥***W***(*g*_tgt_ – *g*_*k*_ – ***J***(*u*_*k*+1_ – *u*_*k*_))∥_2_. In this formulation *u* and *g* encompass all *n*_i_ nonbonded interactions. In some situations, it is desirable only to update some potentials and ignore the match of some RDFs. An example would be a situation where one wants to add a bead type to an existing CG model. In this case, only the new intermolecular potentials should be updated, and the old ones should be fixed. This procedure is used in Section 4.3 to optimize only the pair potential between different bead types in molecular mixtures. To achieve this, eq 3 can be adapted. All potentials that should not be updated have their respective columns in the Jacobian removed. All RDFs that shall not be considered targets have their respective rows removed from the Jacobian and the residuum vector. For the HNC methods, the full Jacobian always has to be constructed first and then reduced. For IMC, interactions can be left out from the start when generating the IMC matrix.

### 2.8 Constraints

When using the Gauss–Newton algorithm, each iteration *k* consists of a linear least-squares problem, because eq 4 can be rewritten as a minimization of
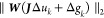
22with *Δg*_*k*_ = *g*_*k*_ – *g*_tgt_, which is solved for *Δu*_*k*_. The potential update can be required to meet certain criteria by constraining the solution of the least-squares problem to fulfill
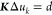
23The number of rows in the matrix ***K*** and vector *d* equals the number of linear constraints. Generally, for any function ϕ(*r*, *g*, *u*) of the pair distance, RDF, and pair potential, a constraint can be derived. To show this, ϕ is expanded up to the first order
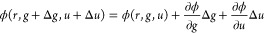
24Here, ϕ(*r*, *g*, *u*) is the value of the function in the current iteration, i.e., ϕ_*k*_. If we define ϕ(*r*, *g* + *Δg*, *u* + *Δu*) as ϕ_tgt_, reorder eq 24, and use *Δg* ≈ – ***J***Δ*u*, we obtain
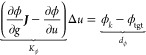
25This form gives us a row in ***K*** and an element in *d*.

From this general formulation, we derive the pressure constraint for multibead systems. The function ϕ is in that case the virial pressure *p* with the integral over the *n*_cut_ grid points of *r* in discrete form
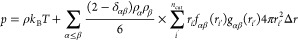
26The factor 2 – δ_*αβ*_ arises because the double sum over β and α is usually unrestricted in the virial. The index *i*′ runs over grid points shifted by half the grid spacing Δ*r* from the usual grid. The values of the pair distance, RDF, and force at those in-between grid points are defined by , , and , respectively. For the pressure expansion, we neglect  in eq 25. This is a good approximation for all but the first few iterations, after which the changes in the RDF are usually much smaller than the changes in the potential. The elements of row *K*_*p*_ in ***K*** are obtained as
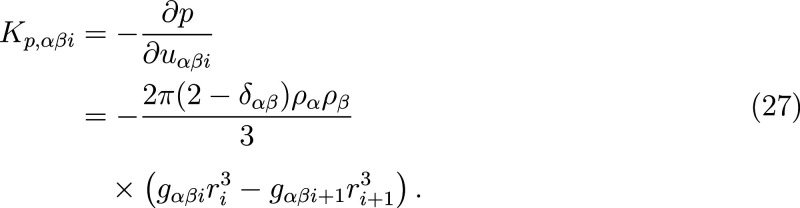
27The value of the element of *d* is given by *d*_*p*_ = *p*_*k*_ – *p*_tgt_. This formulation for the pressure constraint is different from that in our previous papers,^19,20^ where the Gauss–Newton algorithm would update the force instead of the potential. Another difference is that in the previous papers, *p*_tgt_ was used in the constraint formula while we here assume *Δg* is small and therefore use *g*_*k*_.

Next, we introduce a constraint that aims to match the Kirkwood–Buff integral (KBI) of the target RDF. The KBI between two species α and β in a homogeneous system can be written in discrete form as
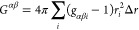
28Here, *i* is the index running over distances which in theory should go to infinity and in practice goes up to *n*_res_. Applying eq 25 on eq 28, we obtain
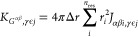
29and
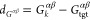
30*G*_*k*_^*αβ*^ and *G*_tgt_^*αβ*^ are obtained from the current and target RDF and eq 28. In a system with multiple interactions, the constraint can be applied to multiple or all interactions.

The intermolecular (cohesive) potential energy of a system determines its vaporization enthalpy. In a system of only bonded and pair interactions, it can be expressed as
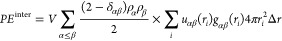
31We note that eq 31 does not explicitly exclude intramolecular pair potentials for large molecules. The formulation given here was only tested for molecules without intramolecular pair potential interactions. We use eq 25, ignore  for the same reason described for the pressure constraint at eq 27, and constrain *PE*^inter^/*N* to obtain a system size independent formulation
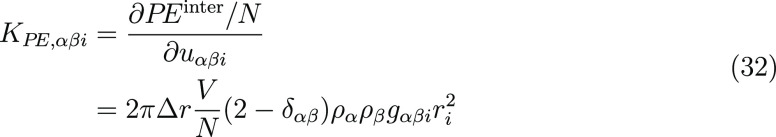
32and
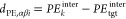
33

We prepend *p*-, *PE*-, or *KBI*- to the method name to denote that the pressure, intermolecular potential energy, or Kirkwood–Buff integrals of the atomistic system are set as constraints to the potential update, respectively. The *KBI*- prefix denotes that all nonbonded interactions have been constrained, so this is not a single constraint but multiple. A surface tension constrained as described for IMC^17^ was not implemented, but we note that our code is easily extensible.

To solve the least-squares problem with constraints, we use a direct elimination algorithm as described in ref (32).

### 2.9 RDF Onset Extrapolation

**Figure 1:**
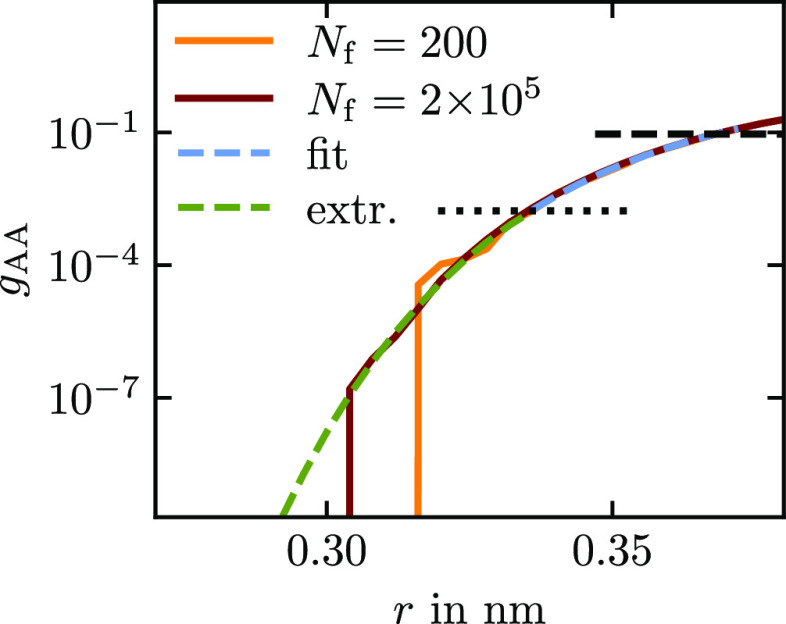
Two A–A RDFs with different samplings were obtained from an OPLS/AA hexane liquid trajectory mapped with a three-bead scheme (see Section 3.2). The system has 3000 molecules, and *N*_f_ is the number of trajectory frames analyzed. Also shown is an extrapolation which was obtained from fitting the potential of mean force of the *N*_f_ = 200 RDF in the region between the dotted and dashed horizontal lines at 0.001 and 0.1 with an exponential function.

Due to the Pauli exclusion principle, atoms never get to be in the same position. This leads to all atomistic RDFs starting with zero. Coarse-grained beads behave similarly if the mapping scheme does not aggregate too many atoms in one bead. Therefore, there is usually an onset region where the RDF goes from zero to nonzero values. This region suffers from poor sampling as few beads ever get to come this close together. The sampling problem becomes visible in a logarithmic plot of the RDF, e.g., in Figure 1. There is visible noise at low values even with long sampling, and below some threshold, all values are zero. The noise and discontinuity can lead to critical problems for coarse-graining algorithms. For example, if the initial potential guess is the potential of mean force (PMF) (same as eq 5), then the repulsive part of the potential can become very noisy or steep, causing instabilities in the MD simulation. Different problems arise for potential updates and are discussed in Section 2.10.

We assume that in the onset region the RDF follows the following asymptotics
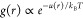
34Equation 34 implies that, up to an additive constant, the PMF matches the pair potential in the onset region if the RDF is perfectly sampled. To extrapolate, we assume that the potential in the repulsive region can be described with an exponential decay. We therefore propose to take −log(*g*), fit it with *f* = *ae*^–*bx*^ + *c* in the well-sampled region, extrapolate into the poorly sampled region, and then take *e*^–*f*^ to obtain the extrapolated RDF. The region for the fit has to be at the onset of the RDF, where eq 34 holds. At the same time, the values have to be reasonably well sampled. We made good experiences with taking the region where the RDF has values between 0.0001 and 0.1 and extrapolating all values that are below 0.0001. In Figure 1, we show an example for an extrapolation of a poorly sampled RDF. It extrapolates the RDF smoothly to very small values. We find it more consistent to extrapolate the RDFs before deriving potentials rather than extrapolating the potentials later. It also simplifies the usage of constraints in the potential update, which get violated when altering the potential after the Gauss–Newton update.

### 2.10 RDF Onset Newton Instabilities

**Figure 2:**
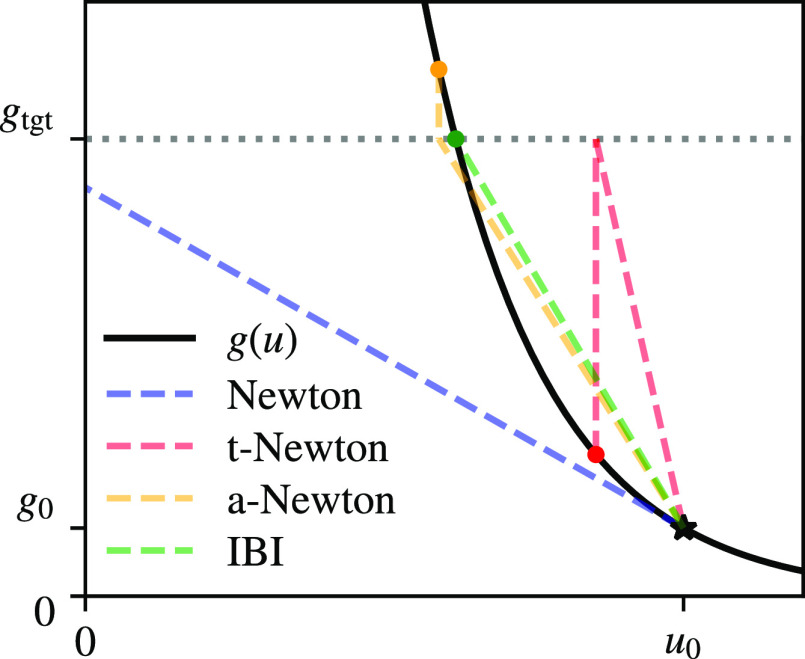
Analytical relation of eq 34 between a single value of the RDF and the potential value at the same distance in the onset region. Starting from potential value *u*_0_, we show different update schemes and their values after one iteration. The star shows the starting point, and the colored dots show *u* and *g* after the update.

Even with the RDF onset extrapolated, as described in the last section, there is a subtle detail that often leads to artifacts in the updated potential obtained from Newton’s methods. The following observation can often be made when using IMC, independent of the sampling. When the current RDF has lower values at the onset region compared to the target RDF, the Newton update will overshoot to a very negative potential. In practice, one will observe deep dips in the repulsive region of the updated potential and small peaks in the RDF onset region. We illustrate the cause of this effect in Figure 2, where we show *g* as a function of *u* at a fixed distance assuming the analytical form from eq 34. The overshoot from *u*_0_ to negative *u* with Newton’s method happens because the derivative at *u*_0_ is very small. In other words, in the onset region, it happens very quickly that current and target RDF have values that are small but orders of magnitude different (e.g., 10^–3^ and 10^–5^), and given the nonlinear relation between *g*(*r*) and *u*(*r*), a simple Newton update easily leads to artifacts. When using IMC, there is little sampling in the onset region leading to additional noise. The t-Newton’s method in Figure 2 gets the derivative from *g*_tgt_ (compare Section 2.6) and leads the potential update which is too small. Note that for *g*_0_ > *g*_tgt_ (not shown) the behaviors of Newton and t-Newton switch. Interestingly, IBI does not have this problem. Instead, this is the region where the IBI method works best, as can be seen in Figure 2. This is to be expected, since an IBI update has the form
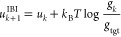
35and if we insert first *g* = *g*_*k*_, *u* = *u*_*k*_ and second *g* = *g*_*k*+1_^IBI^, *u* = *u*_*k*+1_^IBI^ into eq 34, divide the two resulting equations, and apply eq 35, we obtain
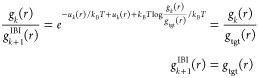
36Therefore, IBI will result in the exact update needed to match the RDF in one iteration under the condition of eq 34. We believe that the stability of IBI played a considerable role in its success in coarse-graining. IBI will always converge well in the onset region and prevent crashes caused by unphysical potentials.

**Figure 3:**
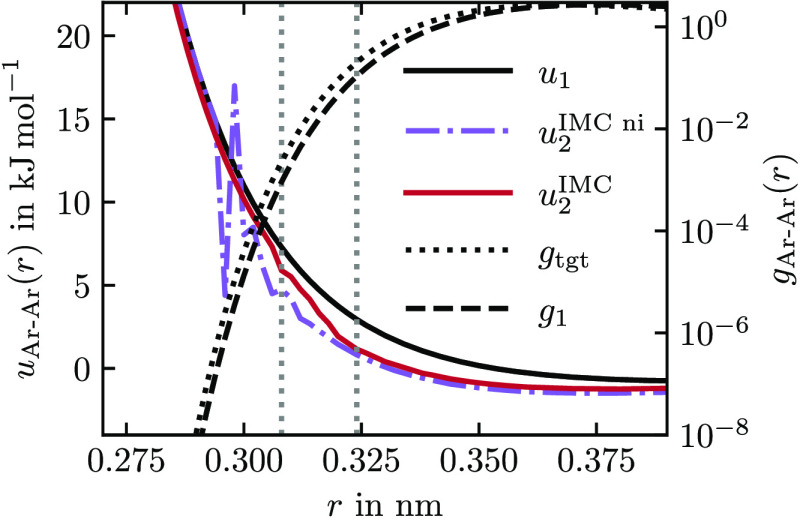
Ar–Ar potential after an IMC update with the nonimproved (ni) and improved IMC Jacobian. Also shown is the potential *u*_1_ before the update which is based on the HNC initial guess. To the right of the graph is the scale for the target and current RDF, which are plotted on a logarithmic scale. Vertical gray dotted lines mark the region in which the IMC matrix has been interpolated.

To retain some of the IBI stability with Newton’s method, we can modify the Jacobian in the onset region
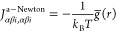
37Here, *g̅* = (*g*_tgt_ + *g*_*k*_)/2 is the average of the current and the target RDF. This option, shown in Figure 2 as a-Newton (a for average), converges satisfactorily in the onset region. We find using a-Newton to be a more consistent fix in comparison to, e.g., having a hybrid scheme where IBI is used in the onset region and Newton elsewhere. The unified formulation with a single Jacobian allows for the straightforward application of constraints later. We have implemented a modification for IMC by changing the Jacobian according to
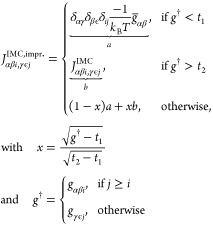
38This formulation is essentially just an interpolation between the diagonal Jacobian from eq 37 and the unmodified IMC Jacobian. The start point and end point of the interpolation are determined by two threshold values *t*_1_ and *t*_2_ that compare against the value of the RDF. The square root was chosen for the interpolation to compensate for the quick growth of the RDF in the onset region. In Figure 3, we show the effect of this modification on the potential update. Without the improvement of the IMC Jacobian, the new potential has a noisy region that can lead to instabilities in an MD simulation. In our experience, if one uses tiny MD time steps and IMC without modification, small peaks in the RDF onset region will show, which do not go away. In previous studies, IBI was often used before IMC, which makes sense due to IBI’s good convergence behavior at the onset region. We have in the past used IMC and IBI alternately to get rid of those artifacts when a single IBI prerun has not worked.^33^ With our improvement to the IMC matrix, the IMC method can be used directly in most cases without any prerun.

For the HNC methods, we modify eq 11 to use the average between the target and current RDF in the diagonal term
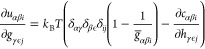
39Because the diagonal term dominates the derivative  in the onset region, this formulation leads to good convergence.

**Figure 4:**
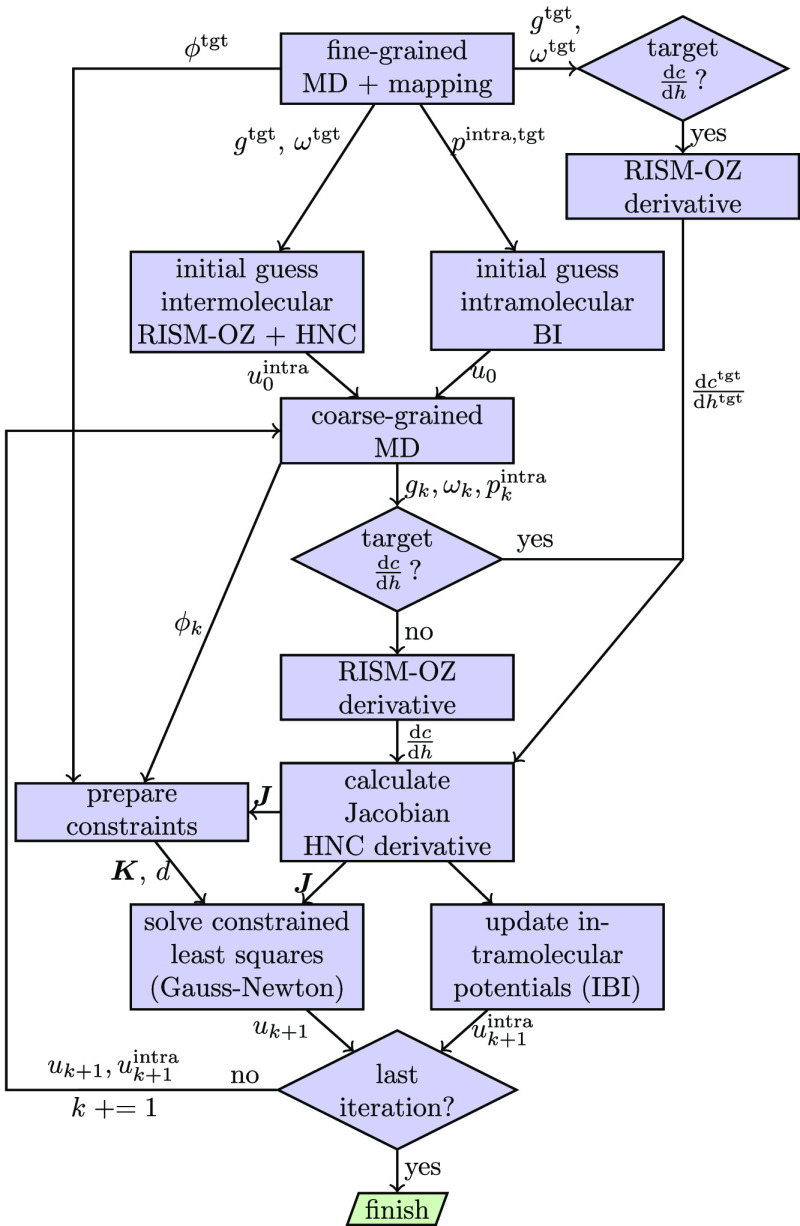
Flowchart showing the iterative process of the HNCGN method with constraints as implemented in VOTCA. Optionally, the derivative  is calculated from the target distributions (t-HNCGN).

In Figure 4, the general process of HNCGN as implemented in VOTCA is shown. The graph encompasses some of the topics discussed in Section 2. It also explicitly mentions the IBI update on intramolecular interactions (bonds, angles, dihedrals). We write, without further details, *p*^intra^ and *u*^intra^ for the probability distributions and potentials of intramolecular degrees of freedom. The formula for the IBI update of bond, angles, and dihedrals is equivalent to eq 6.

## 3 Simulation Details

We simulate two different systems to test the algorithms that were described above. The first is a liquid 1:1 neon–argon mixture at 100 K and 1000 bar. The second is liquid *n*-hexane at 20 °C and 1 bar. To further test our methods and demonstrate their performance at scale, we simulate nine solvents and all their 36 equimolar binary mixtures. The nine solvents are ethanol, 2-butanol, tert-butanol, ethylene glycol, acetone, pyridine, 1,4-dioxane, diethyl ether, and ethyl acetate. They are also considered at conditions of 20 °C and 1 bar

### 3.1 Fine-Grained Simulations

We run atomistic molecular dynamics simulations for all systems to obtain reference properties for the bottom-up coarse-graining procedure. The time step was set to 0.5 fs for all organic systems and 5 fs for the neon–argon mixture. The small time step for the organic systems is needed because hydrogen atoms in flexible OPLS-AA models oscillate with a very high frequency. Molecules were inserted randomly into a box with a density from 10% to 30% lower than the experimental liquid density to obtain an initial configuration. For the insertion, we use GROMACS’ gmx insert-molecules which only inserts a new molecule at positions with little or no overlap with already inserted molecules. Steepest descent energy minimization was performed for 1 × 10^4^ steps. The system was then equilibrated, first under NVT conditions 1 × 10^5^ steps with a stochastic velocity rescaling thermostat with a time constant of 1 ps.^34^ The second equilibration is under NpT conditions for 2 × 10^6^ steps with an additional Berendsen barostat with a time constant of 5 ps. The production run is either performed in the NVT (neon–argon and hexane) or NpT ensemble (solvents) with a Parrinello–Rahman barostat with a time constant of 10 ps and is simulated for 2 × 10^6^ steps. For the NVT production run, the average box volume from the NpT equilibration is used. Trajectory frames are saved every 10 steps.

For neon and argon, the Lennard-Jones parameters are σ_Ne_ = 2.78 Å, ϵ_Ne_ = 0.28869 kJ mol^–1^, σ_Ar_ = 3.401 Å, and ϵ_Ar_ = 0.978628 kJ mol^–1^.^35,36^ OPLS-AA force field parameters for the organic molecules were generated with LigParGen.^37^ This web service creates OPLS force fields with charge models using localized bond-charge corrections.^38,39^ Charges were slightly adjusted to account for the symmetry and the zero net charge of the molecules. The geometric average is used to obtain σ and ϵ for Lennard-Jones interactions between different atoms. Lennard-Jones potentials are shifted upward to yield zero at the cutoff. All systems are simulated with a total of 3000 molecules in a cubic box with periodic boundary conditions. The fine-grained molecular dynamics simulations are performed with Gromacs 2021.5.^40^

### 3.2 Coarse-Graining

For the neon–argon mixture, the CG beads are positioned at the atoms (unity mapping). Thereby, we can test if the various methods retain the atomistic pair potentials.^41^ The hexane molecules are mapped to an ABA representation where B represents the two central carbon atoms and the attached hydrogen atoms and A beads the ethyl groups at either side. The beads are positioned at the centers of mass of the atoms they represent. The CG hexane model contains two bonds and an angle potential as well as two identical beads and is therefore well suited to test the symmetry-adapted RISM methods based on Section 2.4. Nonbonded interactions between the A beads in hexane are excluded. The nine solvent molecules are mapped to single beads. The trajectory of the fine-grained production is mapped according to those mapping schemes, and the RDFs and (for hexane) bond and angle distributions are calculated. For hexane, the intramolecular density functions ω are also calculated. For the t-IMC method (see Section 2.6), the IMC matrix is calculated at this stage.

After the definition of the mapping scheme, the first step of coarse-graining is the initial potential guess. We mainly use the hypernetted-chain equation (eq 9) which gives a very good initial guess.^20,31^ For its evaluation, the maximum available length of the RDF is used, which can be found in Table 1. The initial guess is then cut at the cutoff and shifted such that it is zero beyond. The HNC initial guess is always calculated with eq 9 using the whole RDF. If a t-HNC method is used (see Section 2.6), at this stage also the derivative  is calculated.

**Table 1:** Maximum RDF Length, Potential Cutoff *r*_cut_, Residuum Cutoff *r*_res_, and RDF Grid Spacing Δ*r* Used in the Coarse-Graining of Different Systems^a^

system	max. length	cutoff *r*_cut_	residuum cutoff *r*_res_	Δ*r*
neon–argon	2.4	0.9	–	0.002
hexane	4.2	1.2	1.6	0.004
solvents	3.2	1.2	1.6	0.008

a The residuum length *r*_res_ is only used in Gauss–Newton methods with constraints and corresponds to the distance up to which the RDF difference is minimized. All lengths are in nm.

At each iteration of the coarse-graining procedure, a CG MD simulation was performed with the current potential. The time step is 5 fs for the neon–argon mixture, 1.5 fs for hexane, and 2 fs for all solvents. The size of the time step depends mainly on the “smoothness” of the CG potentials. We found the values above by trial and error, but they are very conservative to ensure that in each iteration the simulation is stable. In most cases, larger time steps are possible with the final CG potentials, but we did not evaluate this for every molecule. Simulations are performed in NVT using a Langevin dynamics integrator with a friction constant of 2 ps^–1^. The density is chosen to match the density of the fine-grained production run. The CG systems have the same number of molecules as the fine-grained systems. We run the CG-MD for 2 × 10^6^ steps for constrained Gauss–Newton methods and IMC and 2 × 10^5^ steps for all other methods. The RDFs calculated from 2 × 10^5^ steps were found to be sufficiently smooth. IMC needs more frames per iteration for the sampling of the IMC matrix; otherwise, there is too much noise in the potential update. In Section 4.1, the smoothness of the RDF and the potential update depending on the number of trajectory frames is explored further. The constrained Gauss–Newton methods are run longer to report well-converged properties, but we also successfully tested them with only 2 × 10^5^ steps. The trajectory is saved every 10 steps, and from it, the RDFs, intramolecular density functions, bond and angle distributions, and optionally the IMC matrix and further ensemble properties are calculated. All RDFs are extrapolated in the onset region with the method described in Section 2.9 with thresholds 0.001 and 0.1. Because CG models sample their available phase space much faster, it is sufficient to discard 100 ps from the MD run as equilibration.

Each iteration ends with the calculation of the potential update. For the IMC update, the IMC Jacobian was modified as described in Section 2.10 with the interpolation region between the RDF values of 0.001 and 0.1. For the HNC methods, eq 39 is used throughout the paper. All Gauss–Newton runs used *r*^2^/(*g*_tgt_ + 10^–30^) as a diagonal on the weighting matrix ***W*** to put weight onto the onset and the tail or the RDF. VOTCA has many options for postprocessing of the potential (e.g., smoothing, shifting, extrapolation), but in this work, we did not use them so as not to convolute their effects with the effects of the CG methods. One exception is the bond and angle potentials in the hexane system, which were extrapolated in unsampled regions after each potential update. For the MD runs, the tabulated potentials are interpolated to match the fixed grid size of GROMACS, which is 0.002 nm.

All coarse-graining tasks are handled and performed by VOTCA. The Gauss–Newton scheme with constraints, the RDF onset improvement, and the integral equation methods have been integrated. The CG molecular dynamics simulations within VOTCA are performed with Gromacs 2019.6 (newer versions do not support tabulated potentials).^40^

## 4 Results

### 4.1 Neon–Argon

**Figure 5:**
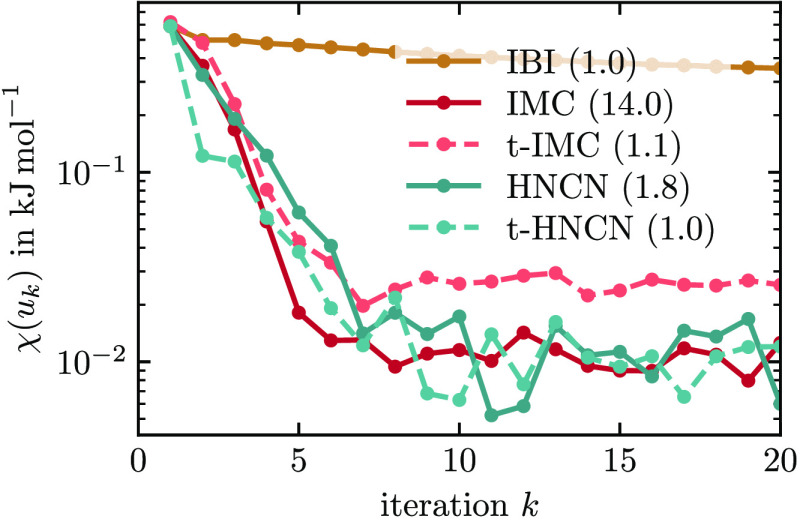
Potential convergence behavior of different methods applied on the neon–argon mixture system. The number in brackets is the time in minutes per iteration on a dual-socket AMD EPYC 7413 machine.

As explained before, the neon–argon system serves as a test case for the methods in this paper, as the true pair potential *u*_ref_ is known. In Figure 5, we show the potential convergence of five different methods measured by the quantity
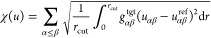
40

The measure χ(*u*) is a sum over all nonbonded interactions and uses the target RDF as a weighting to only measure potential differences in sampled regions. All methods start with the same potential guess and, therefore, the same χ(*u*_1_). With IBI, the potential converges very slowly and does not reach a plateau within 20 iterations. This finding is connected to the underlying low-density approximations of the method as explained in Sections 2.2 and 2.6: The other four methods, IMC, t-IMC, HNCN, and t-HNCN, reach convergence within 10 iterations. Of those, the computational cost per iteration is lowest for t-IMC and t-HNCN, as was explained in Section 2.6.

**Figure 6:**
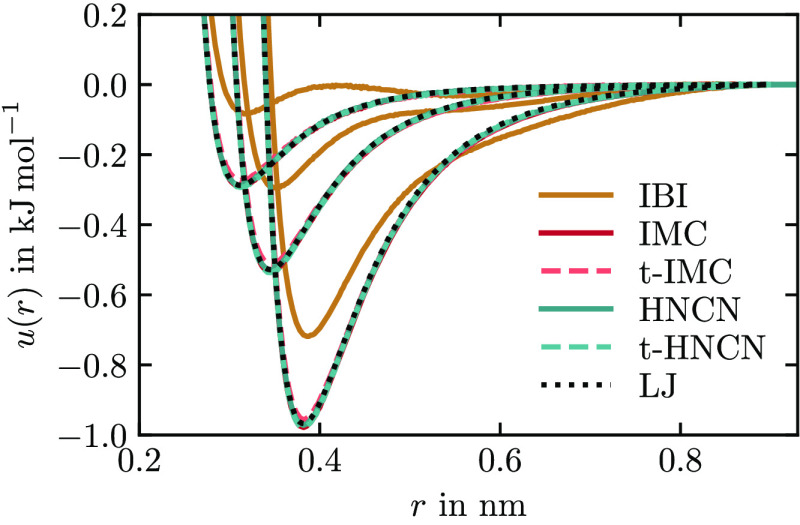
Ne–Ne, Ar–Ne, and Ar–Ar pair potentials (in order of potential minimum position) after 20 iterations of different methods applied to the neon–argon mixture system. Shown as a dotted line is the LJ potential of the reference system.

The height of the plateau is about a factor of 2 higher for t-IMC. This slightly worse convergence is caused by noise in the IMC matrix, which was calculated only once from 2 × 10^5^ atomistic frames. In contrast to IMC, t-IMC always uses the same matrix, and any noise within will therefore persist across iterations. In Figure 6, we show the potentials that result from the different methods from Figure 5. All methods but IBI have converged to the expected potentials after 20 iterations. The potentials of t-IMC have slightly too shallow minima. Note that all potentials produce very similar RDFs (not shown) that are hard to distinguish by the bare eye. Our findings confirm that the HNC methods work well for mixtures. Also, it is demonstrated that by reusing the Jacobian in t-HNCN and t-IMC, coarse-graining can be sped up significantly. There is a small cost in accuracy with t-IMC, while t-HNCN is both accurate and fast.

**Figure 7:**
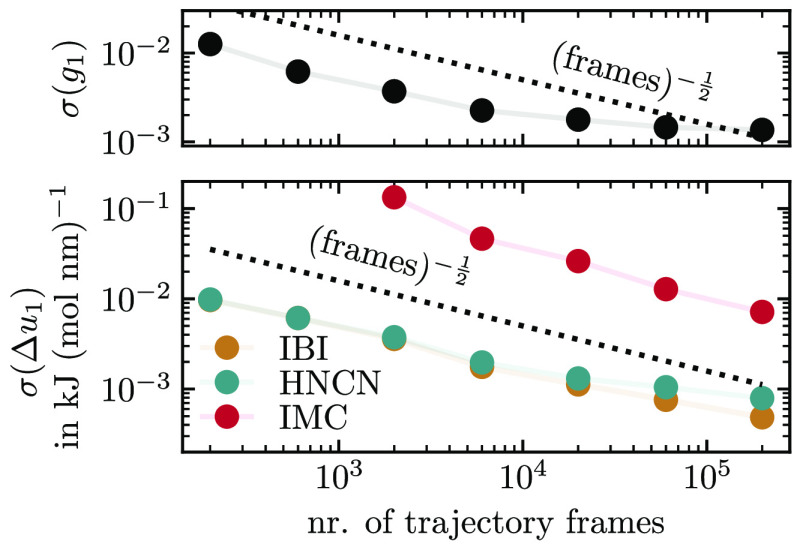
(Top) Noise measure σ(*g*_1_) of the RDF *g*_1_ (based on an MD simulation with the initial guess *u*_0_) in iteration 1. (Bottom) Noise measure σ(Δ*u*_1_) of the potential update Δ*u*_1_ in iteration 1. On the *x*-axis, the number of trajectory frames used for computing *g*_1_ and Δ*u*_1_ is shown. Both panels are log–log plots, and the dotted black lines show the slope of a power function with exponent −1/2.

In Section 3.2, it was mentioned that the use of 10 times more trajectory frames for IMC than IBI and HNCN was necessary because otherwise there is too much noise in the updated potential. Also it was stated that 2 × 10^5^ steps, which corresponds to 2 × 10^4^ frames, yields reasonably smooth RDFs. These statements can be quantified by measuring the noise of the RDF and the potential update for different numbers of frames used to compute the update. We assess the noise by taking the last 20 data points to the left of the cutoff, fitting them with a third-degree polynomial, and calculating the square root of the sum of squares of the distance. This measure is then averaged over the *n*_i_ pair interactions
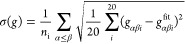
41This same expression can likewise be used to obtain the measure σ(Δ*u*) of the potential update Δ*u*. The results are shown in Figure 7. For both, the RDF and the potential update, the expected relation of the noise measure to the inverse square root of the number of frames is found. The noise of the RDF follows this relationship up to about 6 × 10^3^ frames. Afterward, σ(*g*_1_) decreases slower because the potential *u*_0_ (the HNC initial guess) has some noise itself. Based on this observation, 2 × 10^4^ frames are deemed a sufficient amount of sampling for the RDF. For the potential updates, the noise of the IMC potential is relatively high even for as many as 2 × 10^5^ frames. On the other hand, for IBI and HNCN, the noise is on a similar level, even at as few as 6 × 10^2^ frames. The corresponding 2 to 3 orders of magnitude difference shows how much more sampling is needed per IMC iteration to obtain a smooth potential. The performance difference shown in Figure 5 is based on simulations with 10 times more sampling for IMC. Therefore, by using shorter MD simulations and the HNCN update, the coarse-graining process can be further accelerated.

### 4.2 Hexane

**Figure 8:**
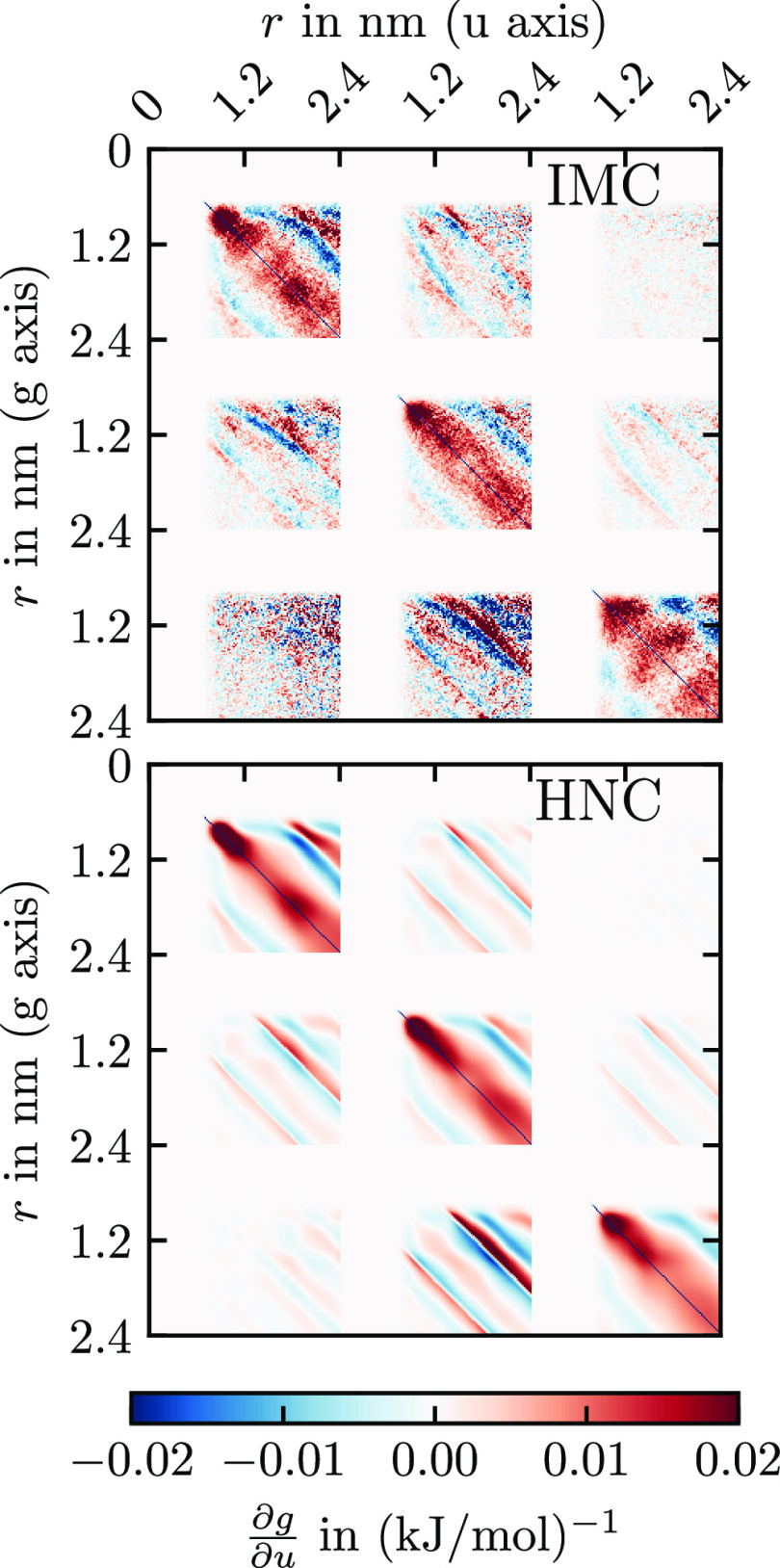
IMC and HNC Jacobian obtained from simulation with the initial guess (for iteration 1). The rows and columns of the visible blocks are related to the interactions A–A, A–B, and B–B.

Before testing convergence on the hexane system, we compare the IMC and HNC Jacobian in Figure 8. We find the IMC Jacobian to be much noisier even though a factor of 10 more frames were sampled for its generation. In general, the two Jacobians have similar structures and intensities. One interesting distinction is the structures of the blocks that are not on the diagonal. There we find that the HNC Jacobian shows a pattern of parallel lines. In the IMC Jacobian, those lines also exist and have similar intensities but are not strictly straight and parallel. This difference is caused by the approximate nature of the RISM equation: It only accounts for the average intramolecular distances but has no information on how they change dynamically when two molecules get close to each other. IMC has that information because it samples the Jacobian from the trajectory.

**Figure 9:**
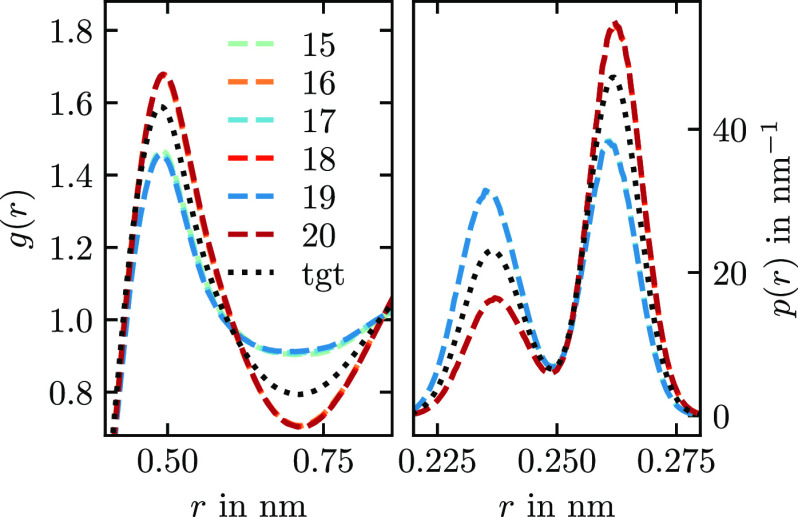
A–A RDF and A–B bond distribution of CG hexane for six iterations of HNCN with simultaneous IBI of bonded potential. The numbers in the legend indicate the iteration *k*.

For coarse-graining hexane, potentials are needed for the angle and the two identical bonds in the CG representation. We make an initial guess by Boltzmann-inverting their target distribution functions. To further refine those bonded potentials, we tried to make IBI updates on them parallel to the update on the nonbonded potentials at every iteration. This, however, led to neither the bonded nor the nonbonded distributions converging, as shown in Figure 9. The problem is that the nonbonded distribution depends to some degree on the bonded potentials and vice versa. However, this connection is not reflected in the potential update method if both are updated independently. What happens is that both interactions are updated as if the other type of potential remains unchanged, which results in an overshoot. This leads to the type of oscillatory behavior that is seen in Figure 9. There are two possible ways to solve this issue. The first would be to derive an integrated update scheme that considers both bonded and nonbonded interactions. This ansatz is possible with the IMC approach but not implemented in VOTCA.^42^ For an HNC-like scheme, one would need the RISM theory to predict bonded distribution functions, and such an equation, to our knowledge, does not exist. A second way is to update nonbonded and bonded interactions alternately. We do this for the hexane system in the rest of the paper and find it to perfectly match the bond and angle distributions after a few iterations.

**Figure 10:**
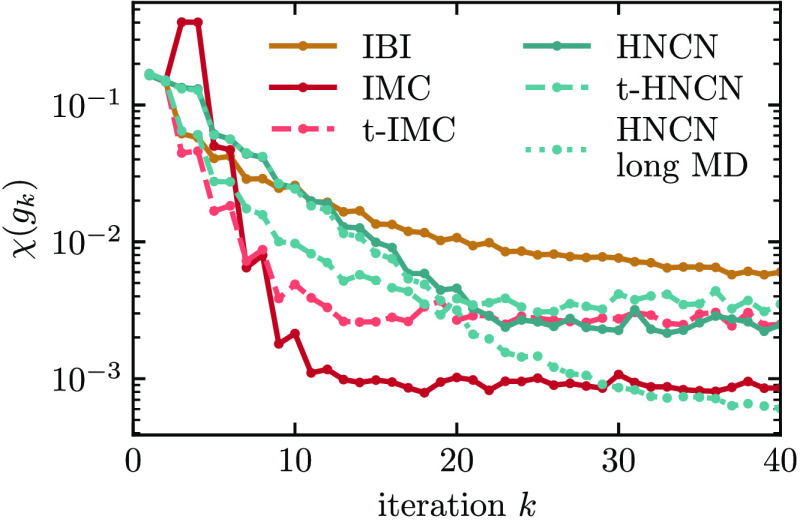
Collective convergence of the three RDFs in CG hexane using different coarse-graining methods. At even iterations, the nonbonded potentials are updated; uneven iterations only update the bonded potentials. The long MD variant of HNCN uses the same number of MD steps per iteration as IMC (2 × 10^6^ instead of 2 × 10^5^).

**Figure 11:**
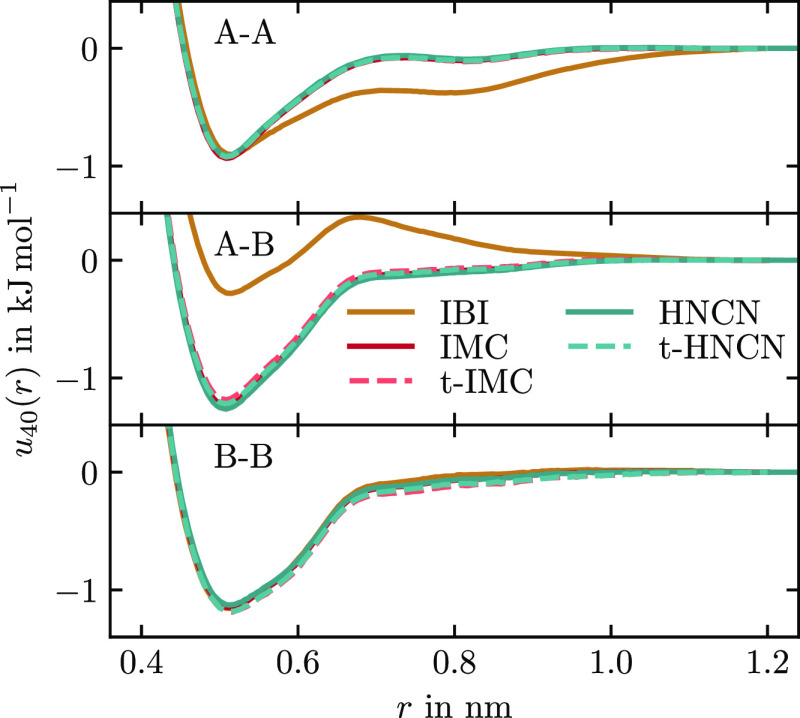
Pair potentials *u*_AA_, *u*_AB_, and *u*_BB_ for CG hexane after 40 iterations of IBI, IMC, or HNCN.

In Figure 10, we see the RDF convergence of different methods applied to hexane. The convergence metric is
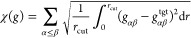
42We find that IBI converges slowest and does not reach a plateau within 40 potential updates (20 bonded, 20 nonbonded). The other two methods converge faster, where IMC is converged after 12 iterations and HNCN after 25. While for neon–argon the convergence of the two methods was similar, here, HNCN is slower. The cause behind the slower convergence is the RISM-OZ equation being a less good approximation for the potential-RDF relation, especially when the molecule is not stiff.^30^ The approximations in RISM-OZ also cause the differences in the Jacobians shown in Figure 8. Besides slower convergence, we find that HNCN converges to a higher plateau than IMC. In principle, one would expect both methods to converge to the same potential due to the Henderson theorem. The difference, therefore, has to come from IMC having a better sampling of the RDF since MD is run ten times longer per iteration. To confirm this hypothesis, the HNCN iterations were rerun with the same number of MD time steps as used for IMC. Also shown in Figure 10, this confirms that HNCN converges to a similar (or even better) precision as IMC with the same amount of sampling. The results from the accelerated t-IMC and t-HNCN methods have similar convergence speeds (iterations to reach plateau) as their unaccelerated counterparts. The accuracies of t-IMC and t-HNCN, however, are reduced compared to IMC and HNCN, respectively. In Figure 11, we show the potential of the last iteration of the same methods from Figure 10. It shows that all methods except IBI find very similar potentials within 40 iterations. The converged potentials fluctuate slightly between iterations which explains the small differences that can be observed.

Next, we tested different Gauss–Newton methods on the hexane system. For the Gauss–Newton scheme, a weighting that puts more weight on the RDF onset and tail is used. As mentioned before, *r*^2^/(*g*_tgt_ + 10^–30^) is used as diagonal on the weighting matrix ***W***. The numerator of the weighting term ensures a good fit of the RDF in the tail region. In combination with *r*_res_ > *r*_cut_, this prevents jumps in the potential at the cutoff which otherwise often occur. The jumps are kept to a minimum by the longer *r*_res_ because they would also lead to a kink in the RDF within the optimized region. The denominator ensures a good fit of the RDF onset region. A small mismatch in this region leads to large fluctuations in the repulsive potential, which can cause instabilities in the MD simulation. The weighting thereby helps to keep the Gauss–Newton iteration stable. The constant 10^–30^ is added to the denominator to ensure the weighting matrix is finite. We note that the software package MagiC^43^ also implements the Gauss–Newton method, but without weighting or constraints.

**Figure 12:**
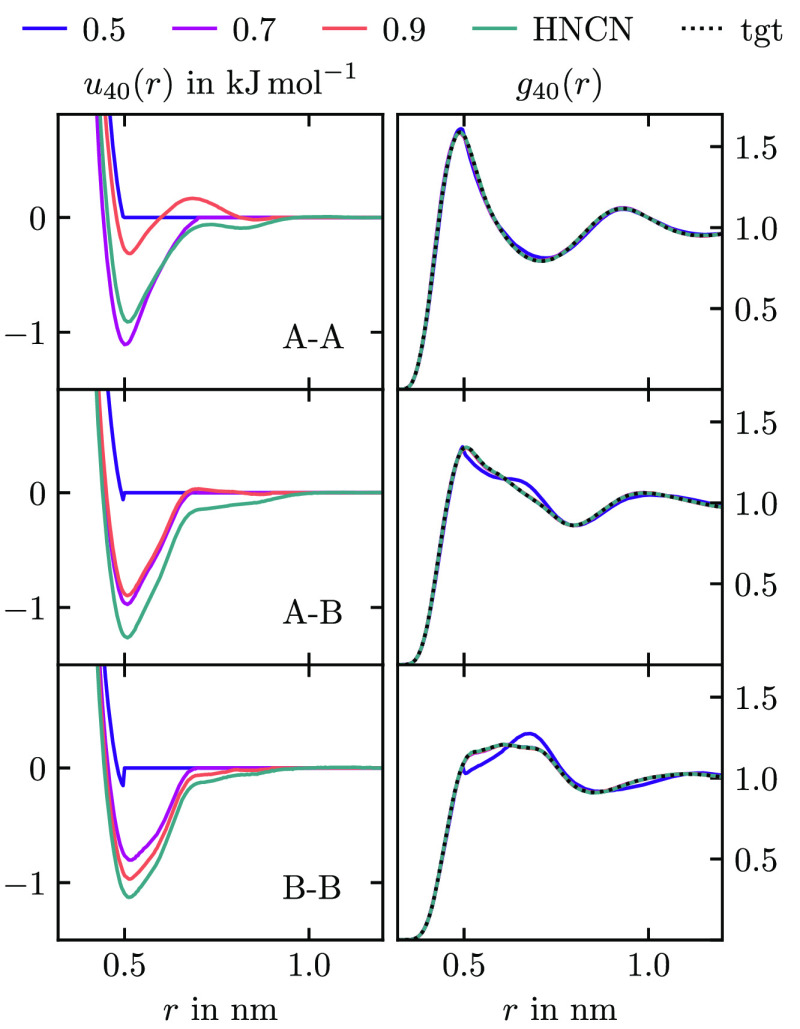
(Left) A–A, A–B, and B–B potentials in CG hexane optimized for 40 iterations to match the RDFs up to 1.2 nm using HNCN and HNCGN. The numbers in the legend are the values of the cutoff *r*_cut_ in nm used for HNCGN. (Right) Resulting RDFs together with the target RDFs.

As a first demonstration of the Gauss–Newton method, we optimize the CG hexane potentials with different *r*_cut_ with a constant *r*_res_ of 1.2 nm. The results are shown in Figure 12. The potentials with *r*_cut_ = 0.5 nm are almost purely repulsive and fail to match the RDFs at larger distances. All other potentials with *r*_cut_ ≥ 0.7 nm possess pronounced potential wells and match the full RDFs well. The changes in the potentials with the cutoff are not predictable and different from just cutting off the tail of the HNCN potential. This shows the need for the Gauss–Newton type scheme when one is interested in parametrizing short-range potentials. Such models are advantageous for their speed in MD simulations.

**Figure 13:**
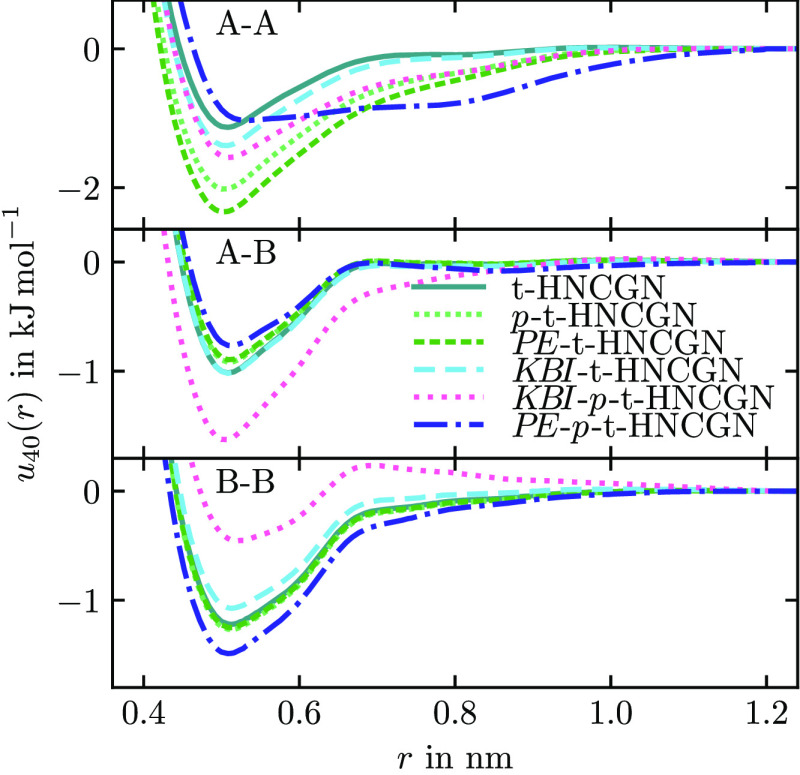
Pair potentials for CG hexane after 40 iterations of t-HNCGN with various constraints.

We test the three constraints for the Gauss–Newton method introduced in Section 2.8 on hexane with *r*_cut_ = 1.2 nm and *r*_res_ = 1.6 nm. The target values are calculated from the atomistic simulation (pressure, intermolecular potential energy) or the atomistic RDF (KBI). The convergence behavior is, in all cases, similarly fast as HNCN, with about 25 iterations to the plateau. In Table 2, we show the resulting properties of the obtained models. We use χ(*g*) as a gauge of the RDF similarity but note that it is not precisely equivalent to the measure minimized by the Gauss–Newton method with weighting. The results show that all properties are reproduced if they were constrained in the potential update. Also, combinations of constraints succeed in reproducing the target values. This is remarkable since it is not directly possible to combine p-IBI with KB-IBI, which both use a ramp correction.^44^ By using the two ramp corrections alternately, it has been possible to achieve a compromise but not a match of the two properties.^45^ We can also see the expected negative impact on the match of the RDFs, as the algorithm gives preference to the constraint. The impact varies between constraints and is the smallest for the KBI constraint and the largest for the combination of the intermolecular potential energy and the pressure constraint. The resulting potentials are shown in Figure 13. They show all very similar structures with different potential well depths. The notable exception is *PE*-*p*-t-HNCGN, which shows a distinctly broader potential well for the A–A interaction. This deviation from the typical form seems necessary to satisfy both constraints.

**Table 2:** Several Properties of Coarse-Grained Hexane Models Obtained from the Gauss-Newton Method with Different Constraints after 40 Iterations^a^

						
	*p*(bar)	*PE*_inter_(kJ mol^–1^)	*G*_AA_(Å^3^)	*G*_AB_(Å^3^)	*G*_BB_(Å^3^)	χ(*g*) ×1000
target	1.0	–30.4	–207.4	–193.3	–201.9	–
t-HNCGN	1075.5(196)	–12.4(3)	–208.0(1)	–194.0(1)	–202.0(2)	1.0(1)
*p*-t-HNCGN	2.2(20)	–25.3(1)	–205.7(1)	–191.4(1)	–199.5(2)	2.9(1)
*PE*-t-HNCGN	–404.1(51)	–30.4(1)	–204.2(1)	–189.8(1)	–197.9(4)	4.2(1)
*KBI*-t-HNCGN	766.3(288)	–16.0(4)	–207.4(1)	–193.3(1)	–202.1(4)	1.7(3)
*KBI*-*p*-t-HNCGN	–0.1(24)	–25.7(1)	–207.3(1)	–193.3(1)	–202.2(3)	3.5(1)
*PE*-*p*-t-HNCGN	2.0(21)	–30.4(1)	–207.7(1)	–191.9(1)	–200.1(2)	11.5(1)

a The numbers are averages of the last six iterations, and the uncertainties reported are the standard deviation of the six values. The last column gives a measure of the mismatch between the obtained and target RDFs as defined in eq 42.

Constraining the KBI between different beads of the ABA hexane molecule was done as a demonstration of our method. Physically, it is not very meaningful, since for a longer integration range *G*_AA_, *G*_AB_, and *G*_BB_ are expected to converge to the same value. This was not the case for *r*_res_ = 1.6 nm. With mixtures, a KBI constraint can be useful in obtaining models that are more transferable between different concentrations.^45^

We note that so far, we have shown a selection of successful attempts at applying constraints. However, we sometimes found constrained HNCGN and IMC-GN not to converge or fail by generating unphysical potentials. Two examples are as follows: (I) We did not succeed in converging a hexane model with all three constraints. (II) We found it not possible to generate a single-bead water model based on the SPC/E RDF that would match KBI and pressure, similar to the setup of Wang et al., who tried with p-IBI.^44^ We use the all-atom definition of the pressure for the CG system but note that alternative definitions exist, often with density-dependent terms.^46,47^ It may be that some of them can be used to obtain CG models that match more thermodynamic observables.

### 4.3 Organic Solvents

**Figure 14:**
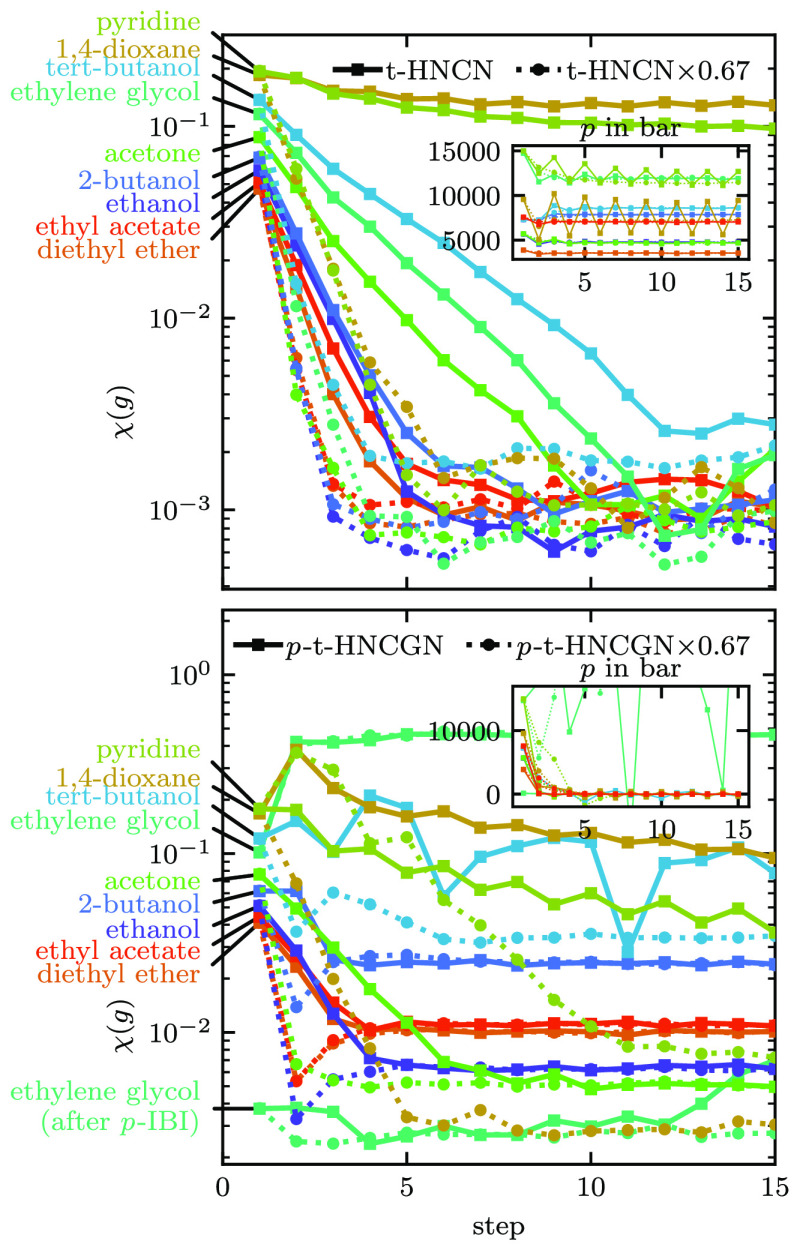
Convergence of the RDF for all pure solvents. The insets show the convergence of the pressure with the same color scheme. In the legend, × 0.67 denotes the scaling factor of the potential update.

In this section, we test our methods on many different systems to learn more about their applicability. We coarse-grain nine organic molecules into single beads. Two different methods are used, t-HNCN and *p*-t-HNCGN, to obtain two models, one which optimally matches the structure and one which has the right pressure and best-possible structure. The convergence behavior is shown in Figure 14. We find that for two molecules, pyridine and 1,4-dioxane, the t-HNCN method does not converge but enters some kind of oscillatory behavior between two potentials that both do not reproduce the RDFs. In the plot, this results in prolonged decay of χ(*g*). For three further molecules, 2-butanol, ethylene glycol, and acetone, the convergence is also significantly slower than for the other molecules. The χ(*g*) value from the initial guess is also the highest for those five molecules. With the *p*-t-HNCGN method, four of those five molecules also show slow or no convergence within 15 steps. The other molecules converge to different χ(*g*) values higher than for t-HNCGN because of the pressure constraint. Note that for the *p*-t-HNCGN method, the convergence is sometimes lower at early iterations, where the pressure is not yet matched.

**Figure 15:**
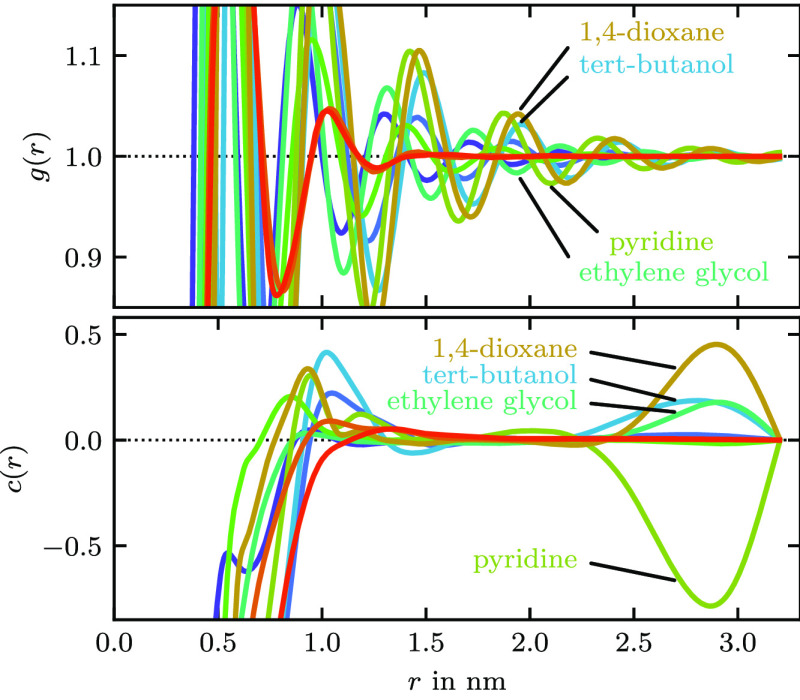
RDFs *g* (top) and direct correlation functions *c* (bottom) calculated from eq 15 for the nine pure solvents. Four lines have been labeled; colors are the same as in Figure 14. For *r* = 0 nm, *c*(*r*) reaches values between −60 and −15, and therefore, only a sector is shown.

To further investigate the convergence problems of some molecules, we show the direct correlation function in Figure 15. The shown functions are the same that were used in the computation of the initial potential guess with eq 9. We see that four of the five molecules with slow convergence also have a local extremum in their direct correlation function at large *r*. This is not typical as *c* is expected to be short-ranged. The way the direct correlation function is computed (eq 15), it is assumed to have decayed to zero at half the length of the RDF input. For all molecules, the RDF was calculated up to 3.2 nm. For those molecules indicated in Figure 15, *c* has not decayed to zero at 1.6 nm. The local maxima are then caused from applying eq 15 on finite RDF data. The intensity of the oscillations in the tail of the RDF, also shown in Figure 15, correlates to the occurrence of the local maxima in *c*. We did not further investigate what sets those four molecules apart, but we note that they have the highest experimental melting points of the nine solvents. Ideally, one would calculate longer RDFs and compute the potential guess and  from them. Nevertheless, we can also work with the RDF data available since an approximate Jacobian can still be used to reach convergence. Based on the oscillatory behavior of the potential, we know that the potential update is overshooting. We, therefore, scale the update by 67% and append × 0.67 to the method name to denote that scaling was used. The results are also shown in Figure 14. This leads to the convergence of both RDF and pressure in all but one cases: *p*-t-HNCGN × 0.67 for ethylene glycol. For this one molecule, we pregenerate a pair potential by using p-IBI for 15 iterations.^44^ Starting from the last p-IBI potential, we find that *p*-t-HNCGN × 0.67 converges.

**Figure 16:**
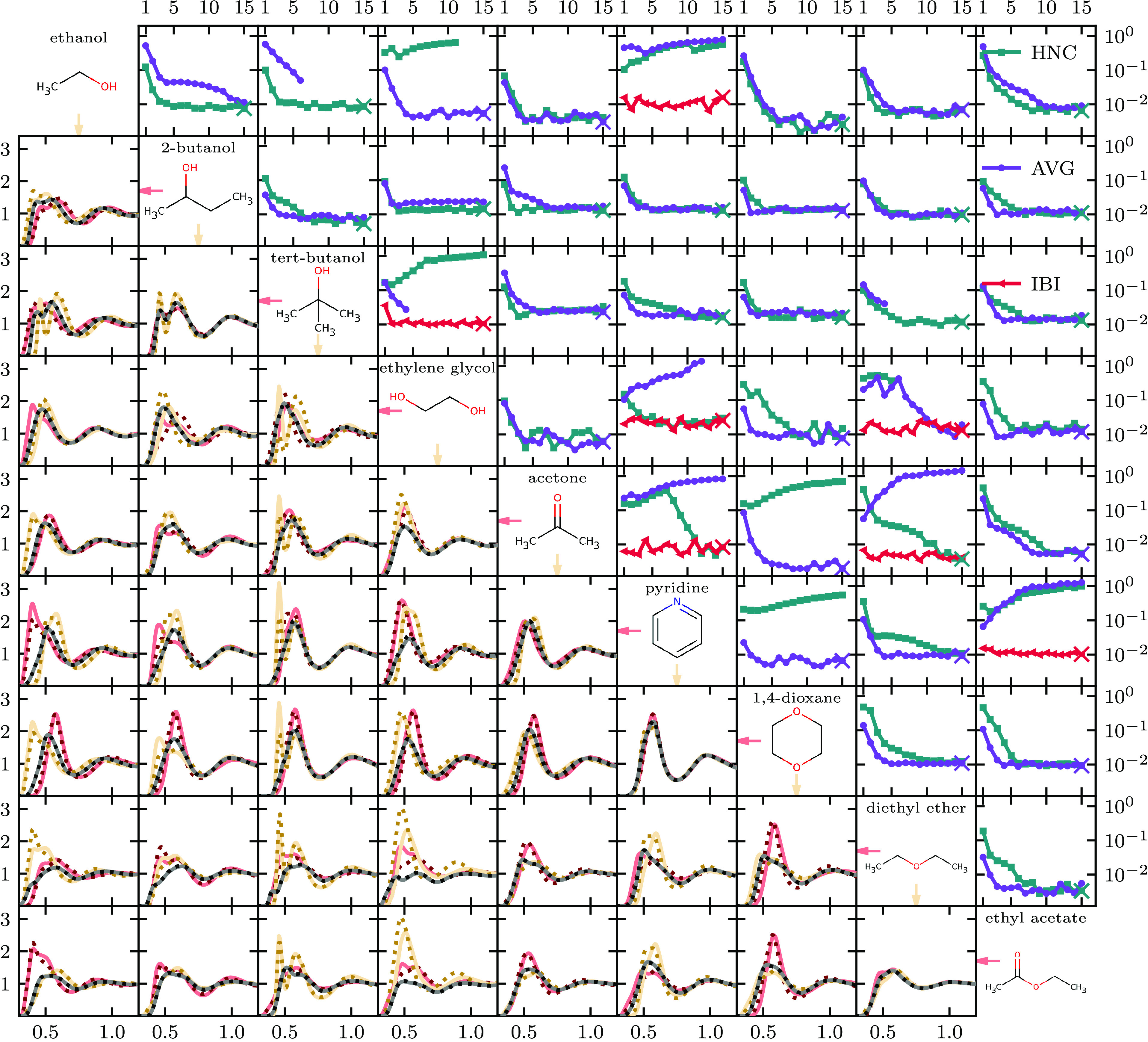
(On diagonal) The nine organic solvents that were coarse-grained into single beads. (Above diagonal) Convergence plots for all mixtures that show χ(*g*_AB_) plotted against iteration number. In all cases, the t-HNCGN × 0.67 method is used to optimize the potential *u*_AB_ to match the RDF *g*_AB_, where A and B are the components of the mixture. Different colors and markers indicate different initial guess methods for *u*_AB_. For some mixtures, 15 IBI iterations were performed (not shown), and Newton’s method started from the IBI outcome. The x in each convergence plot marks the run that has the best RDF match at iteration 15. (Below diagonal) RDFs for all mixtures at iteration 15 of the coarse-graining run that is marked with an x above the diagonal. The distance *r* between beads on the *x*-axis is in units of nm. The gray, red, and yellow lines are *g*_AB_, *g*_AA_, and *g*_BB_ between the molecule in the same row and column on the diagonal, respectively. Their atomistic target RDFs are shown as dotted lines in black, dark red, and dark yellow.

Furthermore, we parametrize pair potentials for all equimolar binary mixtures of the nine solvents. We decided to keep the pair potentials between alike molecules that were obtained from the coarse-graining of the pure solvents. Therefore, for each mixture, only the mixed interaction potential between the two molecules is optimized as described in Section 2.7. The target distribution is the RDF of that same interaction up to *r*_res_ = 1.6 nm. We use the t-HNCGN × 0.67 method without constraints, for which the derivative  is determined once from all three target distributions. In Figure 16, we show the convergence behavior and the resulting RDF for the mixtures. Different ways to generate an initial guess for *u*_AB_ were tested. First, we generated an HNC potential guess from eq 9 and only used it for *u*_AB_. This worked for about 80% of the mixtures and would lead to convergence, while the rest would not. For some mixtures, e.g., ethanol + ethylene glycol, the mixture would phase separate at iteration 1 with the initial guess potentials. The following updates were not meaningful, as we assume homogeneity for the integral equation methods. The phase separation of the mixture is caused by the inconsistent combination of different potentials. In other cases, e.g., ethylene glycol + tert-butanol, it would not converge, but not due to phase separation of the mixture. Rather, the fluid would partially freeze due to the potential *u*_AB_ becoming very attractive. As an alternative initial guess for *u*_AB_, we tried (*u*_AA_ + *u*_BB_)/2, denoted as AVG for average in Figure 16. This led to similar outcomes as with the HNC initial guess: About 80% of the mixtures would not converge. After those two attempts, seven of 36 mixtures had not clearly converged and required a better initial guess. For those seven systems, we performed 15 preiterations of IBI with the HNC initial guess and a potential update scaling of 67%. The final potential was then used and subsequently run for 15 HNCGN × 0.67 iterations. We find that IBI often has converged the RDF rather well, and the following Newton iterations have almost constant χ(*g*). Applying Newton iterations after IBI ensure that the potential is converged, since the space of potentials yielding similar RDFs is large. The reached convergence for all 36 mixtures shows the wide applicability of our approach, and convergence problems can be attributed to the problem of finding an initial guess to which we have proposed three different methods. We have not performed tests on the transferability of our new potentials, but note that with all mixtures converged, one could simulate a system with any composition of the nine molecules.

## 5 Conclusion

We have made progress on three important aspects of structural coarse-graining. First, we have derived and demonstrated how to obtain a fast iterative scheme with integral equation theory for arbitrary molecular systems. It converges similarly fast as IMC for CG systems without bonds and about half as fast if the CG molecule has intramolecular degrees of freedom. By reusing the Jacobian, the cost per iteration is close to that of IBI (which requires far more iterations) and enables the fast derivation of CG models. The reusage of the Jacobian also works for IMC, resulting in an accelerated IMC method. Second, we have investigated the instabilities of Newton methods in the RDF onset region. Due to the exponential relation between RDF and potential in this region, Newton’s methods tend to produce artifacts. We provide a modification for the HNC- and IMC-Jacobian that improves the stability of the methods significantly. This removes the need for regularization or preiterations in most cases. Third, the Gauss–Newton formulation of the inverse problem allows for shorter cutoffs in the CG model and thermodynamic constraints to the potential update. We demonstrate how constraints can be derived if they are a function of pair distance, pair potential, pair force, and RDF. This is demonstrated for pressure, Kirkwood–Buff integrals, and intermolecular potential energy. The possibility of applying several constraints simultaneously is demonstrated. Finally, we have successfully applied our methods to a large number of organic solvents and their mixtures to demonstrate stability and speed that enable consistent and fast derivation of bottom-up CG force fields.

## Appendix: Symmetry-Adapted Distribution Functions

From the analysis of the trajectory, we obtain the distributions *h*_*αβ*_(*r*) and *e*_*αβ*_(*r*) already averaged over the indistinguishable beads of type α and β, but not yet symmetry adapted. The distribution function *e*(*r*) is the intramolecular RDF. Both *h*(*r*) and *e*(*r*) can be obtained with our extension to VOTCA’s csg_stat tool. Here, we derive the equations needed to obtain *H*_*αβ*_ from *h*_*αβ*_ and Ω_*αβ*_ from *e*_*αβ*_.

In Section 2.5, we introduced the matrix ***h***′ which has individual rows and columns for each bead. In contrast, ***h*** has one RDF per bead type combination. The elements in each block of ***h***′ are equivalent to a single element in ***h***

43

Here, *h*_*αβ*_ is an element of ***h***, and ***h***′^*αβ*^ is a block of ***h***′. ***H*** and ***h*** are already of the same dimensionality, but the weighting that comes from the transformation matrix ***T*** still has to be applied. The first column of the transformation matrix (see Section 2.4) has its elements given by

44

If we apply the transformation, insert eq 43, and calculate the top left element, we obtain

45

Here, we have used the property (***T***^α^)^−1^ = (***T***^α^)^T^ of orthonormal matrices. The same prefactor of (*n*_α_*n*_β_)^1/2^ applies for the relation between the elements in ***C*** and ***c***.

For the intramolecular density function, the starting point is the definition of ω′_*kl*_ between beads *k* and *l*. It is most easy defined in Fourier space

46

Here, *e*′_*kl*_(*r*) is the intramolecular RDF between bead *k* and *l*. The density ρ_*l*_ is the number density of bead *l*. The first term in eq 46 represents the delta-peak of the reference atom, which is included in ω′ but not in *e*′. The elements of **ω** are the average over a row in a block from **ω**′ (see ref (22))

47

The same relation holds between ***e***′ and ***e***. Combining eqs 46 and 47 and using *n*_β_ρ_*l*_ = ρ_β_, we obtain

48

Applying the transformation matrix to a single block of **ω**′ and using eq 48 gives

49
